# The semantic of a pandemic: Are cardiovascular patients dying “with” or “from” COVID-19? Reflections from a case report

**DOI:** 10.1097/MD.0000000000025072

**Published:** 2021-03-05

**Authors:** Massimo Mapelli, Valentina Mantegazza, Piergiuseppe Agostoni

**Affiliations:** a Centro Cardiologico Monzino IRCCS; b Department of Clinical Sciences and Community Health, Cardiovascular Section, University of Milan, Milan, Italy.

**Keywords:** cardiovascular risk factors, COVID-19, elderly, pandemic

## Abstract

**Rationale:**

Northern Italy has been particularly hit by the current Covid-19 pandemic. Italian deceased patients have a mean age of 78.5 years and only 1.2% have no comorbidities. These data started a public debate whether patients die “with” or “from” Covid-19. If on one hand the public opinion has been persuaded to believe that Covid-19 infection has poor outcomes just in elderly and/or fragile subjects, on the other hand, hospitals are admitting an increasing number of healthy young patients needing semi-intensive or intensive care units.

**Patient concerns:**

At the end of March 2020, a 79-year-old patient (M.G.) was admitted to the emergency department of our hospital with a 5 days history of fever, dyspnea, and cough. He was known for hypertension and coronary artery disease with a previous coronary artery stenting. Both the comorbidities were carried out without complications and the patient was previously asymptomatic and in good health. At admission, he was febrile and showed signs of respiratory failure with hypoxia and hypocapnia at blood gas analysis.

**Diagnosis:**

The day after, he was tested for SARS-CoV-2 with a real-time reverse transcriptase-polymerase chain reaction assay of nasopharyngeal swab, which turned positive and a chest CT-Scan was consistent with the diagnosis of interstitial pneumonia.

**Interventions:**

He was treated with i.v. diuretics, paracetamol, prolonged noninvasive ventilation (CPAP), and empiric antibiotic therapy on top of his chronic treatment.

**Outcomes:**

A treatment with heparin and corticosteroids was started; however, he developed irreversible respiratory failure. Invasive ventilation was not considered appropriate due to his comorbidities, low chances of recovery, and intensive care unit overcrowding. The patient died 9 days after admission.

**Lessons:**

Health conditions that are most reported as risk factors are common cardiovascular diseases that can be managed in modern clinical practice. Through a brief illustrative clinical case, we would like to underline how Covid-19 can be per se the cause of death in patients that would otherwise have had an acceptable life expectancy.

## 1 Introduction

Northern Italy has been particularly hit by the current Covid-19 pandemic. Italian deceased patients have a mean age of 78.5 and only 1.2% have no comorbidities. These data started a public debate whether patients die “with” or “from” Covid-19. If on one hand the public opinion has been persuaded to believe that Covid-19 infection has poor outcomes just in elderly and/or fragile subjects, on the other hand hospitals are admitting an increasing number of healthy young patients needing semi-intensive or intensive care units. Moreover, the health conditions that are most reported as risk factors are common cardiovascular diseases that can be managed in modern clinical practice.

Through a brief illustrative clinical case, we would like to underline how Covid-19 can be per se the cause of death in patients that would otherwise have had an acceptable life expectancy.

## 2 Case report

At the end of March 2020, a 79-year-old patient (M.G.) was admitted to the emergency department of our hospital with a 5 days history of fever, dyspnea, and cough. He was known for hypertension and coronary artery disease with a previous coronary artery stenting in 2018. Both the comorbidities were carried out without complications and the patient was previously asymptomatic and in good health.

At admission, he was febrile and showed signs of respiratory failure with hypoxia and hypocapnia at blood gas analysis. He was treated with i.v. diuretics, paracetamol, prolonged noninvasive ventilation (CPAP), and empiric antibiotic therapy on top of his chronic treatment, including inhibitors of the renin-angiotensin-aldosterone system. The day after, he was tested for SARS-CoV-2 with a real-time reverse transcriptase-polymerase chain reaction assay of nasopharyngeal swab, which turned positive and a chest CT-Scan was consistent with the diagnosis of interstitial pneumonia. A treatment with heparin and corticosteroids was started; however, he developed irreversible respiratory failure. Invasive ventilation was not considered appropriate due to his comorbidities, low chances of recovery, and intensive care unit overcrowding. The patient died 9 days after admission.

## 3 Discussion

**Figure 1:**
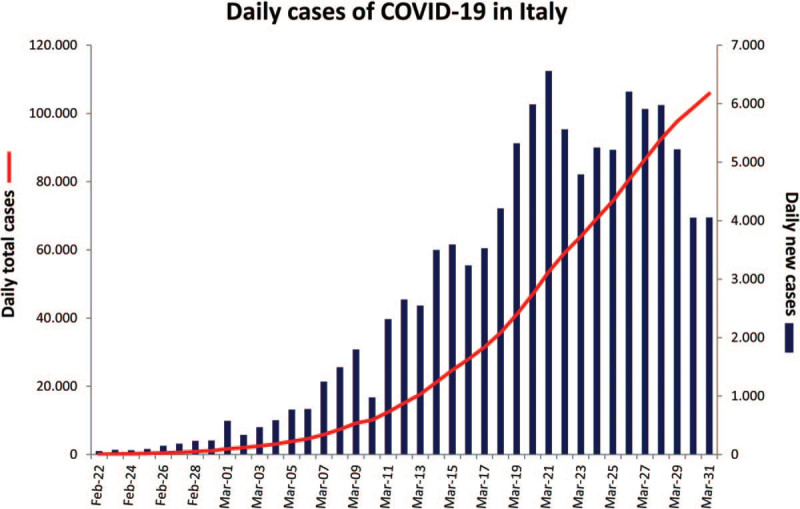
Cases of Covid-19 across Italy during the “first wave” of the pandemic in March 2020. Shown are the official statistics of confirmed daily cases of new coronavirus disease in Italy, according to the National Health Commission as of March 31, 2020. The red line represents daily total cases (new and previous diagnoses); the blue histogram represents daily new cases.

In the last months, a novel coronavirus disease (COVID-19) outbreak has spread rapidly worldwide. At the moment, it is a major international concern, as confirmed by the World Health Organization that has declared it a pandemic. Northern Italy has been particularly hit by this extraordinary public health crisis. Several hospitals, including ours which is a tertiary referral center specialized in heart diseases, have suspended all nonurgent activities to devote resources to the fight against the epidemic. During last spring, we faced the so-called “first wave” of the pandemic and the entire country has been locked down. According to the Istituto Superiore di Sanità, there were 105,792 confirmed cases (including 8% of health workers) as of March 31st (Fig. 1), overcoming China in death number (12,428 vs 3312, respectively). Moreover, it has been suggested that both confirmed cases and total deaths were probably underreported in Italy as well as in other countries.^[1]^ This could be ascribed to the lack at that time of a full standardized protocol for COVID-19 infection diagnosis and low sensitivity of most of the available tests.^[2]^ In addition, in the first phase, only a small proportion of symptomatic patients were tested, as most of them were managed at home until the onset of severe symptoms. These factors, along with only partial compliance with some of the norms of social distancing and protection from contagion, meant that the lockdown strategies put in place in the country worked only partially. Moreover, the increasing attention of the mass media to the pandemic, instead of correctly informing the population about the risks of contracting the disease, has sometimes polarized the debate by giving contradictory messages (e.g., on the use of personal protective equipment) that have contributed to its spread.^[3]^ Although waiting for a large-scale vaccination and in the absence of specific drugs effective against the virus, it is essential to emphasize the importance of strategies aimed at containing infections, especially in subjects at high risk.

Mean age of the Italian victims was 78.5 (median 80) years and a report from the Istituto Superiore di Sanità on March 20th highlighted that only 1.2% of deceased patients had no comorbidities.^[4]^ Specifically, ischemic heart disease was present in 30.1%, atrial fibrillation in 22.0%, hypertension in 73.8%, and diabetes in 33.9%. The first reports from China on hospitalized COVID-19 patients^[5,6]^ similarly showed a high prevalence of pre-existing cardiovascular conditions. Elderly people with comorbidities, especially those with hypertension, coronary heart disease, or diabetes, were more likely to be infected with SARS-CoV-2 and, in case, to develop severe symptoms.^[7]^ Specifically, Huang et al^[6]^ reported that among patients with COVID-19 infection requiring hospitalization in intensive care unit, 23% had underlying cardiovascular diseases, and 15% were affected by hypertension. All these data raise a major issue in the unfortunate case of death of subjects with cardiovascular comorbidities: has the patient died due to his/her underlying condition or due to pneumonia? In other words, has he/she died “with” or “from” COVID-19? The first hypothesis would mean that the infection is considered just as an event breaking a labile clinical balance, as seasonal flu could be in fragile patients. On the contrary, SARS-CoV-2 has widely proved to be a more aggressive disease, potentially leading to life-threatening clinical conditions even in young healthy subjects. If patients died “with” COVID-19, the scientific community should question the benefits of recent medical advances as well as our therapeutic approach to the most common cardiovascular diseases. Actually, the truth is that major progresses have been made in cardiovascular care in the last decades leading to an increasing number of individuals with well-controlled health conditions. New technologies are available in the modern era, which have dramatically increased our knowledge on previously unknown or poorly understood cardiovascular diseases. The emergence of new molecular and genetic testing, machine learning models, engineering simulations, and 3-dimensional imaging software are just some of the new techniques that, together with novel treatments, have improved our diagnostic and therapeutic capacity. Elderly patients with cardiovascular diseases have unprecedented life expectancy and experience an acceptable or good quality of life, even thanks to the advent of new treatment strategies, such as early implantation of ventricular assist devices, mini-invasive surgery, transcatheter valvular and vascular procedures, advances in pharmacological and nonpharmacological interventions (i.e., recent introduction of innovative disease-disease-modifying therapies such as sacubitril/valsartan or SGLT2 inhibitors, implantable cardioverter defibrillator, or cardiac resynchronization therapy in heart failure patients). Moreover, a good control of hypertension, always called into question in these days as a major risk factor in COVID-19 infection, has dramatically improved the prognosis of hypertensive patients, leading to a survival rate comparable to healthy subjects of the same age.

Our patient was a 79-year-old man with significant comorbidities that would have definitely caused his death in the 80s. Instead, he was still an independent man after successful coronary stenting and, before his last hospitalization, he also used to take care of his grandchildren. There are no doubts that he died *from* COVID-19, being his infection-related acute respiratory failure much more than just a straw that broke the camel's back.

## Acknowledgments

The author would like to thank Novartis Farma SpA, Italy, for its unconditional support.

## Author contributions

**Conceptualization:** Massimo Mapelli, Valentina Mantegazza.

**Supervision:** Massimo Mapelli, Piergiuseppe Agostoni.

**Writing – original draft:** Massimo Mapelli, Valentina Mantegazza.

**Writing – review & editing:** Massimo Mapelli, Valentina Mantegazza, Piergiuseppe Agostoni.
